# The Treatment of Certain Forms of Neuralgia

**Published:** 1882-02

**Authors:** 


### THE TREATMENT OF CERTAIN FORMS OF NEURALGIA.

Dr. C. Lange (*Hospitals-Tidende*, 2nd series, bd. 7) in the course of an elaborate article on neuralgia, reports that he has successfully made use of an old, but apparently little known remedy in the treatment of sciatica. The remedy consists in cauterizing the helix of the ear with Vienna paste. In five out of eight cases thus treated, either great improvement or complete cure resulted. The disadvantage of this method of treatment is that the ears are disfigured as the result of the cauterizations. In supra-orbital neuralgia, Dr. Lange has obtained good results from the constant current. He calls attention to the intermittency of the attacks, but does not believe that they are generally connected with malarial infection. In galvanization of the supra-orbital nerve, the anode is placed over the track of the nerve.

